# The contagion effect of heterogeneous investor groups

**DOI:** 10.1371/journal.pone.0292795

**Published:** 2023-10-18

**Authors:** A-Young Park, Gabjin Oh

**Affiliations:** Division of Business Administration, Chosun University, Gwangju, Republic of Korea; Università Cattolica del Sacro Cuore Sede di Piacenza e Cremona Facoltà di Economia: Universita Cattolica del Sacro Cuore Facolta di Economia e Giurisprudenza, ITALY

## Abstract

This paper suggests an alternative approach to measuring systemic risk in financial markets by examining the interconnectedness among heterogeneous investors. Utilizing variance decomposition and a trading database from the Korea Stock Exchange spanning 2002-2018, we find that systemic risk, as quantified by total connectedness based on microlevel investor activity, intensifies during both domestic and global financial crises. In addition, our analysis indicates that retail investors, often termed noise traders, are pivotal contributors to the propagation of financial shocks. We also find that portfolios constructed by the sensitivity of total connectedness yield additional returns. This study could enhance our understanding of the contagion effect by incorporating the investor perspective, and the findings could offer valuable insights for policy-makers and regulators.

## Introduction

The Global Financial Crisis (GFC) of 2007-2008 and the collapse of Silicon Valley Bank highlighted the systemic risks inherent in modern financial markets. To maintain effective regulation, it is important to understand the contagion effect based on the complex interdependence of financial markets due to cross-border financial activities [1–6]. While the prediction of future market dynamics is a popular research topic, there is no consensus regarding how to measure systemic risk [7–11].

Existing research on network theory concentrates on risk propagation [12–14]. Moreover, most of the studies measuring systemic risk have examined stock volatility or portfolio similarities among financial institutions rather than examining investor behavior [6, 15–21]. Given the complexity of financial systems, our study aims to consider heterogeneous investors and the ramifications their interactions, such as misinformation and herding behavior. These factors have recently been exemplified by events such as GameStop Saga and the rise of cryptocurrencies (e.g., Runa). Therefore, a bottom-up approach is needed to explore the behavior of investors or companies, thereby enhancing our understanding of systemic risk [22–27].

In light of these challenges, our study introduces an alternative approach for quantifying systemic risk through investor-based connectedness. We explore the interconnectedness among heterogeneous investors via their trading activity, indirectly examining the psychological factors that influence market behavior. Our research contributes to the literature by incorporating a microlevel analysis and adopting an information view among investors in the financial market [28, 29]. To the best of our knowledge, this aspect of measuring systemic risk—i.e., based on investor activity—has not been addressed in the literature.

To assess systemic risk at the microlevel, we employ variance decomposition based on aggregated daily trading values on the Korea Stock Exchange from 2002 to 2018 [30]. This technique allows us to dissect the interconnectedness among heterogeneous investor groups, serving as a channel for propagating structural shocks through the financial system. This approach is advantageous because it captures both the intensity and directionality of connections. The investor groups are classified into nine distinct categories and are represented as the nodes in directed and weighted networks. In these networks, the edges denote their influence on the unpredicted errors of the aggregated trading values of commonly traded securities.

The summary of our empirical evidence is as follows. Systemic risk measured by heterogeneous investors increased during both domestic and global financial crises. While existing studies have focused on measuring systemic risk among financial institutions, this study takes an approach by introducing an investor-based connectedness measure tailored to capture the characteristics of the Korean stock market. This finding enriches the literature on how both positively and negatively correlated signals are transmitted through information linkages among traders [24, 31]. Furthermore, our results reveal that retail investors play a pivotal role in propagating negative shocks, which is consistent with studies suggesting that retail trading imbalances can induce co-movement in stock returns [32]. Lastly, we find a negative relationship between differences in total connectedness, as measured via variance decomposition, and expected returns. This finding is consistent with the literature that has shown the role of net individual trading in explaining stock returns [33].

This study has several implications. By identifying retail investors as key drivers of systemic risk, we provide insights for policy-makers and regulators regarding potential vulnerabilities in financial markets. Investors and portfolio managers may also benefit from our findings by optimizing their risk assessment strategies to include investor-based connectedness measures. This microlevel approach to measuring systemic risk suggests further examination that could integrate both the macro and micro perspectives. It remains unclear how investor activity contributes to risk propagation before prompting early policy action.

The remainder of this paper is organized as follows. Section 2 describes the source of our data and the variance decomposition for measuring connectedness. Section 3 and Section 4 present the main findings related to market stability and price formation. Section 5 provides the conclusion.

## Materials and methods

This section describes the methods that measure connectedness among investor activities in our analysis and is divided into four subsections. First, we describe the data and explain how we use the variables to estimate the forecast error causality among investor groups. In the second subsection, we define connectedness using the vector autoregression (VAR) and variance decomposition method (VDM). In the third subsection, we suggest the role of investors in terms of determining market stability. In the fourth subsection, we consider empirical asset pricing models to analyze the relationship between connectedness as a systemic risk and the expected return as a risk premium.

### Data description

Our dataset contains daily trading values of buyers and sellers for 1,154 common stocks listed on the Korea Composite Stock Price Index (KOSPI) from January 1, 2002, through December 30, 2018. We obtain the closing price, stock return, and trading volume of investors’ stocks from FnGuide. Trading volume is an important proxy for market liquidity, and we use it to estimate the forecast error causality among investor groups. The trading value of each investor is the stock price multiplied by the trading volume at a daily frequency. To classify the investors, we group them into nine distinct types based on their classification in the Table 1 from FnGuide, excluding overlapping groups: Foreign (FO), Retail (RE), Banks (BA), Insurance (IS), Investment (IV), Pension Funds (PF), Financial Investment (FI), Other Financial (OF), and Other Corporate (OC).

**Table 1:** Investor types classified by the FnGuide.

Investor types	Description
Foreign (**FO**)	Foreigners registered with given unique numbers with the Financial Supervisory Service.
Retail (**RE**)	An investor holding shares in a personal account.
Banks (**BA**)	Commercial banks, local banks, special banks (Korea Development Bank and Export-Import Bank of Korea), the National Agricultural Cooperative Federation, the National Federation of Fisheries Cooperatives, and the National Federation of Livestock Cooperatives under the Banking Act.
Insurance (**IS**)	Life insurance, non-life insurance, guarantee insurance and reinsurance companies under the Insurance Business Act.
Investment (**IV**)	Investment management, investment companies, and mutual funds as defined under the Securities and Investment Trust Business Act.
Pension Funds (**PF**)	The corporations prescribed by a presidential decree that manage and operate funds established by law (the National Pension, the Teachers’ Pension, and the Public Officials Pension) and those that operate a deduction business under the law (the Military Mutual Aid Association, the Credit Cooperatives Federation, the Construction Mutual Aid Association, and the Korean Teachers’ Credit Union).
Financial Investment (**FI**)	A securities company under the Securities Exchange Act, which includes foreign securities firms with a license.
Other Financial (**OF**)	Comprehensive financial and mutual savings banks (mutual credit unions and Saemaeul savings banks).
Other Corporate (**OC**)	Domestic corporations excluding securities companies, insurance companies, investment trust companies and investment companies, banks, comprehensive financial institutions, mutual savings banks, pensions, funds, mutual aid associations, etc.

**Fig 1:**
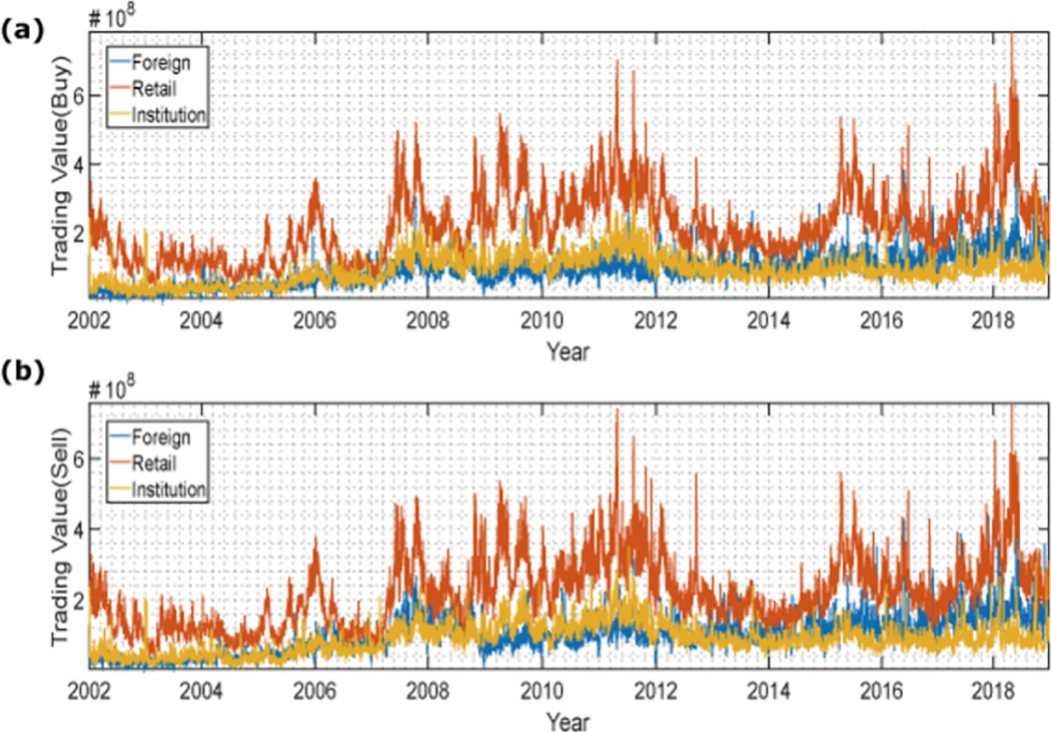
(Color online)Time series of trading value of buy and sell of investor groups. In this figure, we categorize investors into three distinct groups: foreign investors, retail investors, and institutional investors. The latter is an aggregate category that includes the sum of all other types of investors, excluding foreign and retail investors.

We collect the trading values of stocks to create a time series for each investor. Table 2 shows the description of the natural logarithm of aggregate trading value on the buy and sell side among nine investors. Fig 1 depicts the trends in trading values on the buy and sell sides for three investor groups: foreign investors, retail investors, and institutional investors. The latter is an aggregate category that includes the sum of all other types of investors, excluding foreign and retail investors.

**Table 2:** Data description of aggregate investor trading.

Panel A: Aggregate Buy Trade					
Investor Type	Mean	Std. Dev.	Skewness	Kurtosis	JB test
FO	19.03	0.48	-0.27	2.56	70.03
RE	16.35	0.79	-0.53	2.86	194.06
BA	13.50	0.71	-0.07	3.49	29.51
IS	15.91	0.75	0.26	2.85	132.16
IV	18.01	0.70	-0.96	3.44	500.99
PF	15.56	1.02	-0.64	2.34	484.48
FI	16.01	1.11	-1.00	3.35	635.55
OF	14.75	0.61	0.01	3.32	808.66
OC	17.06	0.61	-0.04	2.56	99.80
Panel B: Aggregate Sell Trade					
Investor Type	Mean	Std. Dev.	Skewness	Kurtosis	JB test
FO	19.04	0.48	-0.29	2.60	78.28
RE	16.32	0.76	-0.48	2.80	154.56
BA	13.59	0.73	0.02	3.75	107.74
IS	15.79	0.81	0.43	2.93	228.30
IV	18.09	0.75	-0.99	3.44	558.83
PF	15.53	1.01	-0.64	2.34	488.08
FI	15.88	1.23	-1.06	3.42	760.81
OF	14.85	0.63	0.49	5.05	809.84
OC	17.09	0.63	-0.04	2.58	79.72

This table presents the mean, standard deviation, skewness, and kurtosis of the natural logarithm of trading value for each investor group: Foreign (FO), Retail (RE), Banks (BA), Insurance (IS), Investment (IV), Pension Funds (PF), Financial Investment (FI), Other Financial (OF), and Other Corporate (OC). The sample consisted of 4,203 daily observations of 1,154 companies between 2002 and 2018. The JB test denotes the Jarque-Bera test for a normal distribution, which is significant at the 5% level (critical value 5.97).

We then defined Total Trading (*TT*_*i*,*t*_), Net Trading Imbalance (*NTI*_*i*,*t*_), and Normalized Net Trading Imbalance (*NNTI*_*i*,*t*_) of investor *i* at time *t* as follows.

$$\begin{array}{c} TT_{i,t}=\left(ln\left(Buy_{i,t}\right)+ln\left(Sell_{i,t}\right)\right)-\left(ln\left(Buy_{i,t-1}\right)+ln\left(Sell_{i,t-1}\right)\right) \end{array}$$ (1)

$$\begin{array}{c} NTI_{i,t}=\left(ln\left(Buy_{i,t}\right)-ln\left(Sell_{i,t}\right)\right)-\left(ln\left(Buy_{i,t-1}\right)-ln\left(Sell_{i,t-1}\right)\right) \end{array}$$ (2)

$$\begin{array}{c} NNTI_{i,t}=\frac{ln\left(Buy_{i,t}\right)-ln\left(Sell_{i,t}\right)}{ln\left(Buy_{i,t}\right)+ln\left(Sell_{i,t}\right)}-\frac{ln\left(Buy_{i,t-1}\right)-ln\left(Sell_{i,t-1}\right)}{ln\left(Buy_{i,t-1}\right)+ln\left(Sell_{i,t-1}\right)} \end{array}$$ (3)

where *Buy*_*i*,*t*_ is the aggregate buying trading value of investor *i* at daily frequency *t* and *Sell*_*i*,*t*_ is the aggregate selling trading value of investor *i* at time *t*. Although each aggregate trade is nonstationary, the difference between aggregated trades is stationary to estimate the total connectedness.

We investigate the time-varying influence among investors during major financial crises in the sample period. For instance, credit card numbers rose dramatically to boost consumer spending in 2002 according to the Bank of Korea and Financial Supervisory Service. Specifically, the number of credit cards was 89.3 million in 2001, 104.8 million in 2002, and 95.5 million in 2003. This led to systemic risk in financial markets and had a devastating impact on the real economy in 2003. Likewise, the collapse of Lehman Brothers in 2008 due to imprudent trading of commodities such as mortgage-backed securities (MBSs) and credit default swaps (CDSs) led to global financial turbulence. These crises highlight the role of investor behavior in market stability.

### Connectedness among investors

This subsection represents the measurement of total connectedness based on the causal relationship between investors in the stock market by [30]. We measure the degree of connectedness and the causality among investor groups to examine the market’s cascade effect through closely coupled investors.

As [34] argued, a vector autoregressive (VAR) model used to capture the linear linkages among multiple time series is one of the familiar models in econometrics. All variables in this model are symmetrically considered in a structural sense [35]. The evolution of a set of variables over the sample period is a linear function of only past values. We obtain forecast errors using the estimated VAR model and analyze the response of the shock for each time. we analyze the response of the shock for each time. To do so, we propose using the variance decomposition method (VDM), which is the proportion of the variance of a variable due to each fundamental shock.

To select the lag order, we minimize the Bayesian information criterion (BIC) by setting the lag order p as four, five, and five in TT, NTI, and NNTI, respectively [36]. We also verified that similar results were obtained when using other lag orders, as noted by [30]. We employed a rolling window of 250 days and an H-step of 10 days. Our sensitivity analysis confirmed that the results did not change significantly with different window lengths and H-steps, consistent with previous studies [30]. The choice of connectedness horizon is important because it relates to dynamic connectedness issues rather than purely contemporaneous connectedness.

In this study, we define *Y*_*t*_ as the multivariate time series signals of total trading value of nine investor groups, including Total Trading (TT), Net Trading Imbalance (NTI), and Normalized Net Trading Imbalance (NNTI). We estimate the model using the following equation:

$$\begin{array}{c} A\left(L\right)Y_{t}=\varepsilon_{t} \end{array}$$ (4)

where *A*(*L*) = 1 − *A*_1_*L*^1^ − … − *A*_*p*_*L*^*p*^, and *A*_*p*_ is the matrix of estimators. *p* is the lag order of the model. *ε*_*t*_ represents change in the output of the other variable. *Y*_*t*_ is the multivariate time series signal of the total trading value of various investor groups. To measure the connectedness of each investor, we use the generalized variance decomposition method (GVDM). This method overcomes the limitations of the Cholesky decomposition, which can produce different results when changing variables. The proportion of variables in the prediction errors of the estimator using GVDM can be written as

$$\begin{array}{c} \theta_{ij}^{g}\left(H\right)=\frac{\sigma_{jj}^{-1}\Sigma_{h=0}^{H-1}{\left(e_{i}^{{}^{\prime }}B_{h}\Sigma e_{j}\right)}^{2}}{\Sigma_{h=0}^{H-1}e_{i}^{{}^{\prime }}B_{h}\Sigma B_{h}^{{}^{\prime }}e_{i}} \end{array}$$ (5)

where $\theta_{ij}^{g}\left(H\right)$ is the fraction of variable *i*’s H-step forecast error variance due to shocks in variable *j*, H is the predictive horizon (*H* = 1, 2, 3, …, and we set *H* = 10), $\sigma_{jj}^{-1}$ is a diagonal element of the covariance matrix estimate error, *e*_*j*_ = 1 for the *j*th element and = 0 for other elements, *B*_*h*_ is the coefficient matrix reflecting the shock effect, and Σ is the covariance matrix of the estimated error.

This paper generates the proportion of variables in the disturbance matrix. The directional connectedness from *j* to *i* can be written as the following equation:

$$\begin{array}{c} C_{ij}\left(t\right)=\frac{\theta_{ij}^{g}\left(H\right)}{\Sigma_{j=1}^{N}\theta_{ij}^{g}\left(H\right)} \end{array}$$ (6)

where *C*_*ij*_(*t*) is the pairwise directional connectedness from *j* to *i*, and it measures the fraction of the forecast error variance of variable *i* at time *t* that can be attributed to shocks in variable *j*. In other words, it reflects the contribution of *j*’s shocks to the forecast error of *i*.

The GVDM provides a forecast error table for three datasets within the nine investor categories in the Korean stock market. Each component in the table represents the connectedness among investor groups. Since the above matrix components can be interpreted as the response to other investors’ impacts, the diagonal term represents the effects on investor groups received from themselves. We focus on the contribution of forecast error among investor groups, which allows us to exclude the diagonal components from each investor. Each row of the matrix describes the contribution of others to the own forecast error, while each column of the matrix represents the contribution of own to others. Therefore, we interpret *C*_*ij*_(*t*) as causality for trading behaviors among heterogeneous investor groups.

### Systemwide connectedness

This subsection explains the concepts of directional connectedness and total connectedness (TC), derived from the pairwise directional connectedness matrix *C*_*ij*_(*t*). Directional connectedness captures the quality of investors’ information in the sense that heterogeneous investors contribute to their own and others’ unpredictability. Since our focus is on the influence that each investor exerts on others, we define directional connectedness as the weighted degree of the off-diagonal element of each investor in the matrix *C*_*ij*_(*t*). In this matrix, “To” represents the sum of each investor’s unpredictable error to others and is an outflow to each investor, while “From” represents the sum of the components to each investor from others and is an inflow from each investor. The “Net” value of each investor is calculated by subtracting the “From” from the “To” of each investor. To identify distinct roles in risk propagation, we categorize investor groups with positive (negative) net values as the sources (sinks) of systemic risk. We also introduce a time-varying measure of total connectedness (TC), which is defined as follows.

$$\begin{array}{c} TC\left(t\right)=\frac{\Sigma_{i=1}^{N}\Sigma_{j=1,i\neq j}^{N}C_{ij}\left(t\right)}{N} \end{array}$$ (7)

where *N* is the number of investor groups and *C*_*ij*_(*t*) is calculated by Eq 6. It is equivalent to the average total directional connectedness, such as “From” and “To”, where diagonal terms are ignored. We construct the weighted and directed investor networks using connectedness table *C*_*ij*_(*t*). To analyze the network characteristics according to market status, we construct a network over time with a time window of length Δ*t* = 250 days, moving it by *δt* = 1 day.

In conclusion, Eqs 6 and 7 provide a quantitative measure for analyzing how information flows among heterogeneous investor groups, measuring systemic risk at the investor level. This is a novel approach, as most previous studies have focused on systemic risk at the level of financial institutions or markets as a whole. In addition, our approach enables the identification of specific types of investors who act as significant contributors to the propagation of systemic risk.

### Empirical asset pricing model

There are various types of risk in the financial market. The role of systemic risk in constructing portfolios, which represents the risk-return trade-off, is unclear. In this study, we investigate whether the connectedness at the investor level has a significant influence on the cross-section of expected returns with different sensitivities to systemic risk, which we proxy using the change in total connectedness (Δ*TC*). We follow the concept of [37], where the VIX index is used as a proxy for aggregate volatility.

To estimate the sensitivity of expected returns to changes in systemic risk, we use the following equation with daily data:

$$\begin{array}{c} R_{i,t}=\alpha_{i}+\beta_{i,MKT}MKT_{t}+\beta_{i,\Delta TC}\Delta TC_{t}+\epsilon_{i,t} \end{array}$$ (8)

where *R*_*i*,*t*_ is excess return from the risk-free rate of firm *i* on day *t*, *MKT*_*t*_ denotes the market excess return of the KOSPI, and Δ*TC*_*t*_ is the instrument we use for change in the total connectedness of the total trading dataset. We use the call rate of Korea from the FnGuide as a proxy for the risk-free rate. The coefficient *β*_*i*,*MKT*_ measures the sensitivity of asset *i* to the market factor, while the *β*_*i*,Δ*TC*_ captures the sensitivity of asset *i* to Δ*TC*_*t*_.

We run the regression for all stocks on the KOSPI with more than 16 daily observations within each month. At the end of each month, stocks are classified into quintiles based on the realized value of *β*_Δ*TC*_ loadings on systemic risk over the past month. Firms in the 1st quintile have the lowest *β*_Δ*TC*_ coefficient, while firms in the 5th quintile have the highest *β*_Δ*TC*_ coefficient. Table 5 shows portfolio returns for the quintile portfolio sorted by *β*_Δ*TC*_ over the previous month using Eq 8. We used value-weighted portfolios and do not use multiple factor models for the portfolio beta because controlling other factors can lead to misunderstanding the impact of total connectedness. After constructing a portfolio each month, we calculate the monthly portfolio return, beta, and alpha using the following equations:

$$\begin{array}{c} R_{p,t}=\alpha +\beta_{MKT}MKT_{t}+\beta_{SMB}SMB_{t}+\beta_{HML}HML_{t}+\epsilon_{t} \end{array}$$ (9)

$$\begin{array}{c} R_{p,t}=\alpha +\beta_{MKT}MKT_{t}+\beta_{SMB}SMB_{t}+\beta_{HML}HML_{t}+\beta_{RMW}RMW_{t}+\beta_{CMA}CMA_{t}+\epsilon_{t} \end{array}$$ (10)

where *R*_*p*,*t*_ is the portfolio return generated by *β*_Δ*TC*_ at month *t* − 1. Five factors, MKT, SMB, HML, RMW, and CMA, represent the Fama-French five-factor model’s market, size, value, profitability, and investment factors, respectively. The portfolio *β* captures the sensitivity of the portfolio to the market factor and other systematic factors. The intercept *α* captures any residual return not explained by the model. Traditional models such as the CAPM and the Fama-French five-factor model have not specifically incorporated systemic risk into their frameworks. Our study takes an exploratory approach to investigate whether systemic risk can be considered a type of market risk, potentially offering an extension to these traditional models.

Based on the factor risk model in Eq 8, we hypothesize that assets with higher sensitivity to the change in systemic risk, as measured by a higher value of *β*_*i*,Δ*TC*_, will have lower expected returns. This hypothesis is consistent with the literature on [37], which suggests that stocks with higher exposure to aggregate volatility risk tend to have lower returns. A negative relationship is expected between portfolios based on *β*_*i*,Δ*TC*_ and expected returns, indicating that assets with higher sensitivity to the change in systemic risk earn lower portfolio returns. This approach provides us with insight into the portfolio to generate risk-adjusted returns and the extent to which its returns can be attributed to systematic risk factors.

## Empirical tests of connectedness

In this section, we present the empirical results of measuring systemic risk through investor-based connections, employing both the vector autoregressive (VAR) and variance decomposition (VDM) methods. First, we provide a full-sample analysis that covers the entire sample period. Second, we conduct a rolling sample analysis using specific time windows to examine time-varying trends in systemic risk. Third, we identify the roles of risk propagation among heterogeneous investor groups. Fourth, we suggest new insights for portfolio construction considering systemic risk.

### Full-sample analysis of investor behavior

To examine investor-based connectedness, we perform a full-sample analysis based on trading activity from nine investor groups on the Korea Stock Exchange. We assign an influence score to each investor group, ranging from zero to one hundred. This score is calculated based on the forecast error matrix derived by the VAR and VDM. An influence score of zero indicates no connection with other investors, while a score of one hundred suggests a maximum weight of connection in Table 3. Understanding the influence score is critical for measuring systemic risk, as investor groups with higher influence scores are likely to play pivotal roles during financial crises, either stabilizing or exacerbating systemic risk.

**Table 3:** Full-sample connectedness table, nine-group aggregation.

Panel A: Connectedness among investor groups of Total Trading (TT)										
	FO	RE	BA	IS	IV	PF	FI	OF	OC	From
FO	NA	8.29	5.67	5.80	9.60	5.47	10.88	2.72	3.42	51.84
RE	7.55	NA	7.90	7.49	9.78	6.44	11.49	4.49	4.84	59.97
BA	5.53	8.10	NA	6.81	8.01	5.61	7.83	3.85	5.84	51.58
IS	6.04	8.24	6.98	NA	9.00	6.55	7.80	3.73	3.96	52.31
IV	8.14	8.78	6.68	7.32	NA	8.82	12.37	2.97	5.47	60.53
PF	6.10	6.94	5.96	6.58	10.60	NA	7.69	3.05	4.02	50.93
FI	9.00	10.36	6.88	6.51	12.98	6.33	NA	3.57	3.94	59.55
OF	3.97	6.32	5.53	4.85	4.92	3.93	5.40	NA	3.26	38.18
OC	3.89	5.82	6.82	4.52	7.24	4.17	5.54	2.53	NA	40.53
To	50.23	62.85	52.40	49.87	72.13	47.31	69.00	26.89	34.74	**Total Connectedness**:
Net	-1.61	2.88	0.82	-2.44	11.59	-3.62	9.45	-11.29	-5.79	**465.44/9 = 51.72%**
Panel B: Connectedness among investor groups of Net Trading Imbalance (NTI)										
	FO	RE	BA	IS	IV	PF	FI	OF	OC	From
FO	NA	11.62	0.77	0.19	8.23	2.50	2.80	0.63	4.29	72.90
RE	12.97	NA	0.41	0.45	12.18	1.56	3.66	0.38	0.23	66.70
BA	1.19	0.64	NA	0.69	1.15	0.29	1.18	0.84	0.18	50.80
IS	0.66	0.97	1.22	NA	0.17	0.76	1.17	0.16	0.19	59.05
IV	3.06	18.20	0.96	0.22	NA	2.40	2.22	0.12	0.25	68.74
PF	2.78	1.66	0.30	0.89	1.57	NA	0.38	0.36	0.24	64.85
FI	2.07	4.09	0.89	0.99	2.10	0.54	NA	0.23	0.83	44.39
OF	0.69	0.29	0.88	0.17	0.12	0.27	0.29	NA	0.13	65.22
OC	4.28	1.18	0.07	0.06	0.70	0.40	0.50	0.25	NA	71.43
To	27.70	38.64	5.50	3.66	26.22	8.72	12.21	2.98	6.32	**Total Connectedness**:
Net	-3.33	6.81	-0.67	-1.64	-1.22	0.53	0.48	0.16	-1.11	**131.96/9 = 14.66%**
Panel C: Connectedness among investor groups of Normalized Net Trading Imbalance (NNTI)										
	FO	RE	BA	IS	IV	PF	FI	OF	OC	From
FO	NA	11.15	0.76	0.21	8.31	2.53	2.87	0.58	4.28	30.70
RE	12.43	NA	0.48	0.28	12.29	1.27	3.74	0.42	0.20	31.10
BA	1.17	0.74	NA	0.78	1.14	0.27	1.28	1.94	0.16	6.48
IS	0.80	0.69	1.41	NA	0.26	0.74	1.34	0.16	0.18	5.57
IV	3.11	18.13	0.97	0.36	NA	1.88	2.66	0.13	0.27	27.50
PF	2.88	1.39	0.30	0.75	1.26	NA	0.62	0.40	0.25	7.84
FI	1.94	4.06	0.93	1.13	2.48	0.84	NA	0.24	0.85	12.48
OF	0.64	0.30	1.00	0.16	0.12	0.25	0.33	NA	0.13	2.95
OC	4.37	1.03	0.05	0.08	0.71	0.46	0.52	0.25	NA	7.46
To	27.34	37.49	5.90	3.75	26.58	8.24	13.35	3.12	6.32	**Total Connectedness**:
Net	-3.35	6.39	-0.59	-1.82	-0.92	0.40	0.87	0.17	-1.14	**132.08/9 = 14.68%**

This table lists the connectedness calculated the using aggregated trading value from January 2002 through December 2018. The total connectedness (i, j) represents the percent of the variance of the forecast error of investor i caused by shocks from investor j over a 10-day predictive horizon (h-step). Panels A, B, and C denote total trading, net trading imbalance, and normalized net trading imbalance, respectively. The labels of the first column and row are the abbreviations of the investor groups, e.g., Foreign (FO), Retail (RE), Banks (BA), Insurance (IS), Investment (IV), Pension Funds (PF), Financial Investment (FI), Other Financial (OF), and Other Corporate (OC).

Table 3 displays the connectedness matrix, referred to as the influence score, with Panels A, B, and C each focusing on different datasets: Total Trading (TT), Net Trading Imbalance (NTI), and Normalized Net Trading Imbalance (NNTI). Investor groups are classified into nine categories: Foreign (FO), Retail (RE), Bank (BA), Insurance (IS), Investment (IV), Pension Funds (PF), Financial Investment (FI), Other Financial (OF), and Other Corporate (OC). In Table 3, the *ij*th entry represents the extent to which investor j’s actions contribute to the forecast error variance of investor i. The “From” and “To” labels for each row and column sum indicate the degree to which each investor group is influenced by, or influences, others. The “Net” value for each investor group is calculated as the “To” value minus the “From” value, providing an overall sense of each group’s role in the market’s interconnectedness.

We examine various features of the connectedness matrix presented in Table 3. Previous studies, such as those by [38], have used variance decomposition to assess market connectedness and found results that align with other systemic risk measures, such as marginal expected shortfall and CoVaR [1, 39]. In our table, entries in the “From” column sum up to a maximum of 100%, representing the cumulative connectedness emanating from each investor group, excluding self-connectedness.

We define *Net*_*i*_ as the net connectedness for investor *i*, calculated as “To” minus “From”. A *Net*_*i*_ greater than zero implies that investor *i* primarily disseminates information, while a *Net*_*i*_ less than zero suggests that the investor mainly receives information. Consequently, we categorize investors with a positive *Net*_*i*_ as sources of systemic risk, which transmit structural shocks. Conversely, those with a negative *Net*_*i*_ act as sinks, receiving these shocks. The existence of such sources implies a potential for cascading market failures.

In Panel A of Table 3, we observe the highest pairwise directional connectedness between financial institutions and investment, at 12.37% and 12.98%, respectively. This is followed by the pairwise directional connectedness from financial institutions to retail investors (11.49%) and from retail investors to financial institutions (10.36%). As shown above, financial institutions, investment, and retail investors are more interconnected than others in the market. The investor groups with the highest positive directional connectedness are investment (11.59%), financial investment (9.45%), and retail investors (2.88%). The investor groups that receive the lowest amount of information from others are other financial (−11.29%), other corporate (−5.79%), and pension funds (−3.62%).

In Panel B of Table 3, retail investors, pension funds, financial investment, and other financials show positive directional connectedness. The highest pairwise directional connectedness is from retail investors to investment (18.20%) and from investment to retail investors (12.18%). The second highest pairwise directional connectedness is observed from foreign investors to retail investors (12.97%) and from retail investors to foreign investors (11.62%). The results in Panel C of Table 3 are consistent with those in Panel B.

Our analysis reveals that retail investors significantly influence overall market connectedness, with directional connectedness values of 62.85%, 38.64%, and 37.49% in Panels A, B, and C, respectively. This implies that retail investors could be a key source of negative market shocks. Overall, the total connectedness for the TT, NTI, and NNTI datasets stands at 51.72%, 14.66%, and 14.68%, respectively.

**Fig 2:**
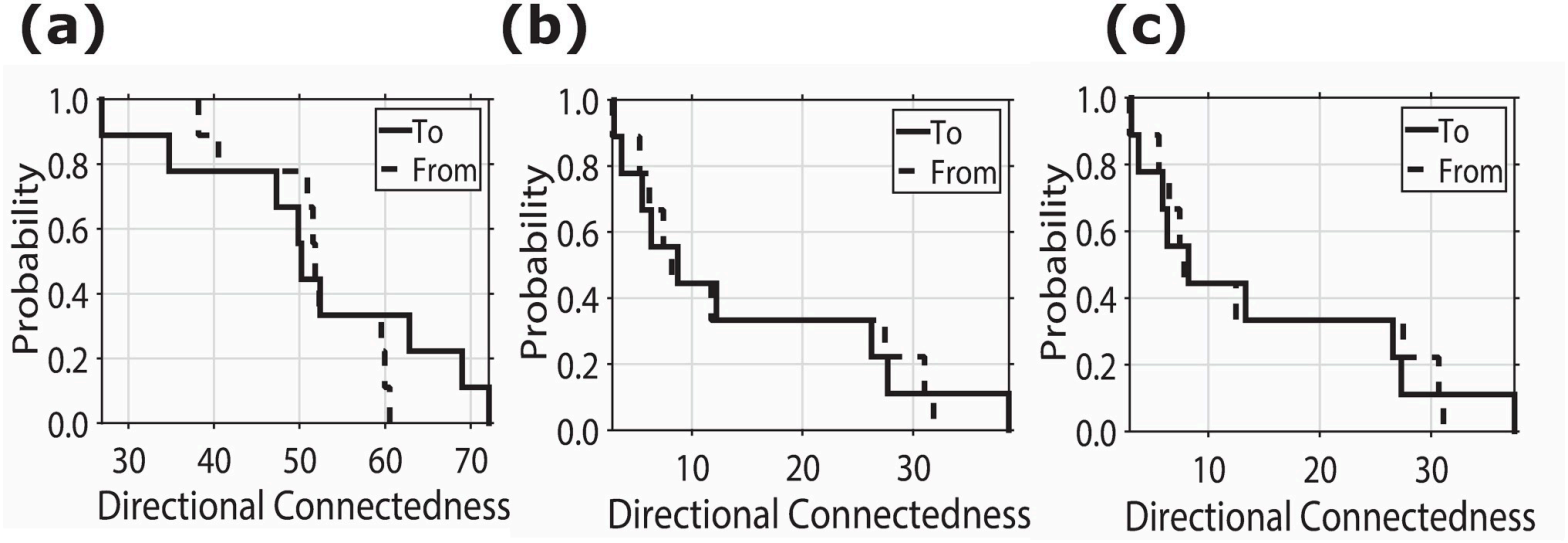
Total directional connectedness. Fig 2(a)–2(c) indicate the total directional connectedness of TT, NTI, and NNTI, respectively. We plot the cumulative distribution function for total directional connectedness “To” others and “From” others. The predictive horizon for the variance decomposition is 10 days.

We consider two ranges of directional connectedness shown in Fig 2(a), where the components are denoted by “To” (solid line) and by “From” (dotted line). The “From” column quantifies the impact of various investors on the variability of the total forecast error. In Fig 2(a), the overall “From” column ranges from 38.18% to 60.53%. Similarly, in Fig 2(b), the ‘From’ column ranges from 44.39% to 72.90%, and in Fig 2(c), it ranges from 2.95% to 31.10%.

It is worth noting that the contributions from other investors to each individual investor are not capped at 100%, allowing for the possibility that entries in the “To” line may exceed 100%. The information contributed by each investor to others varies significantly, whereas the information received from other investors tends to be more uniform. In essence, directional connectedness to other investors is distributed over a broader range when compared to the connectedness originating from other investors, as illustrated in Fig 2.

### Rolling sample analysis of investor behavior

This subsection presents a dynamic analysis of the total connectedness distribution from 2002 to 2018 using a rolling window. We also calculate each investor’s net value each year and identify the investors who lead the information flow in the stock market.

**Fig 3:**
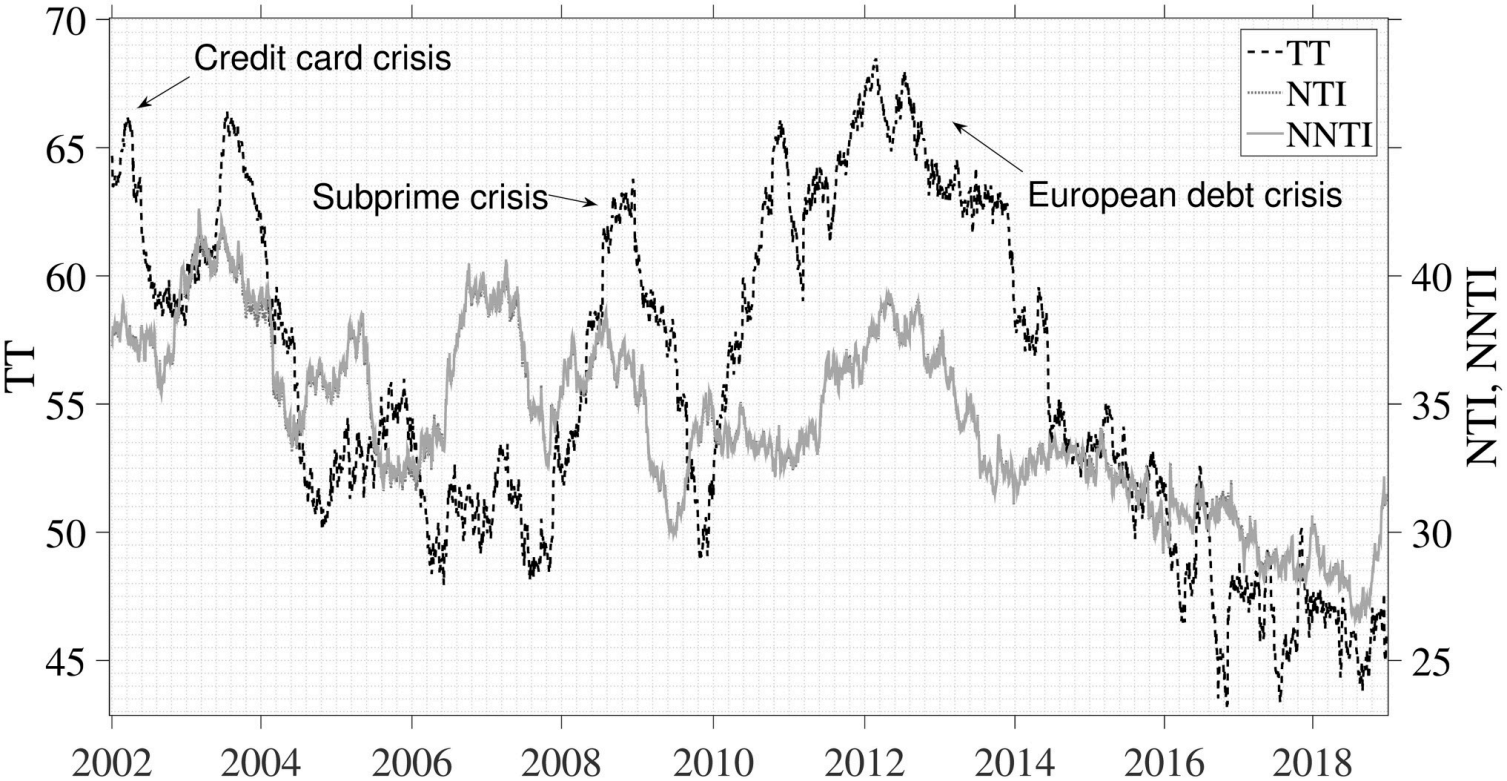
The evolution of total connectedness estimated using a 250-day rolling window. The black line represents total connectedness of TT, and the gray lines represent total connectedness of NTI and NNTI. All three databases show similar patterns of total connectedness, which tend to increase during global financial crises.

Systemic risk at the microscopic level is quantified through connectedness via variance decomposition among nine investor groups using trading values from three datasets: TT, NTI, and NNTI. A 250-day rolling window and prediction horizons of four, four, and five days are employed for this analysis using each dataset. Fig 3 illustrates the total connectedness, calculated as the average of either the “To” or “From” values among investor groups. This metric is derived from Table 2. Note that this measure of total connectedness is capable of detecting time-varying influences among different investor groups. The black line represents the total connectedness of TT, and the gray lines represent those of NTI and NNTI. The x-axis indicates the ending date of each rolling window, and the y-axis delineates the total connectedness of total trading on its left side and the (normalized) net trading imbalance on its right.

The pattern of total connectedness in Fig 3 increases during global financial crises. This result suggests that negative shocks among investors are commonly shared, thereby destabilizing the financial market. As Fig 3 reveals, the average total connectedness of TT is 55.93, but it rose dramatically to 66.03 in March 2002, a surge largely attributed to the widespread use of credit cards in South Korea. The collapse of Lehman Brothers in 2008 had a cascading effect on global markets, exemplified by the total connectedness rising from 48.28 in June 2006 to 53.3 by March 2007. It saw further increases during the European debt crisis in 2010 but started to decline with the onset of the Greek debt crisis in 2012. The end-of-2016 South Korean political scandal also influenced total connectedness, as foreign investors sold significant stock volumes due to heightened market uncertainty. The total connectedness of the three types of datasets follows a consistent pattern with marked increases in 2002, 2008, and 2012. These findings suggest that the KOSPI market’s total connectedness based on trading activity increases during both domestic crises and global financial turmoil.

**Fig 4:**
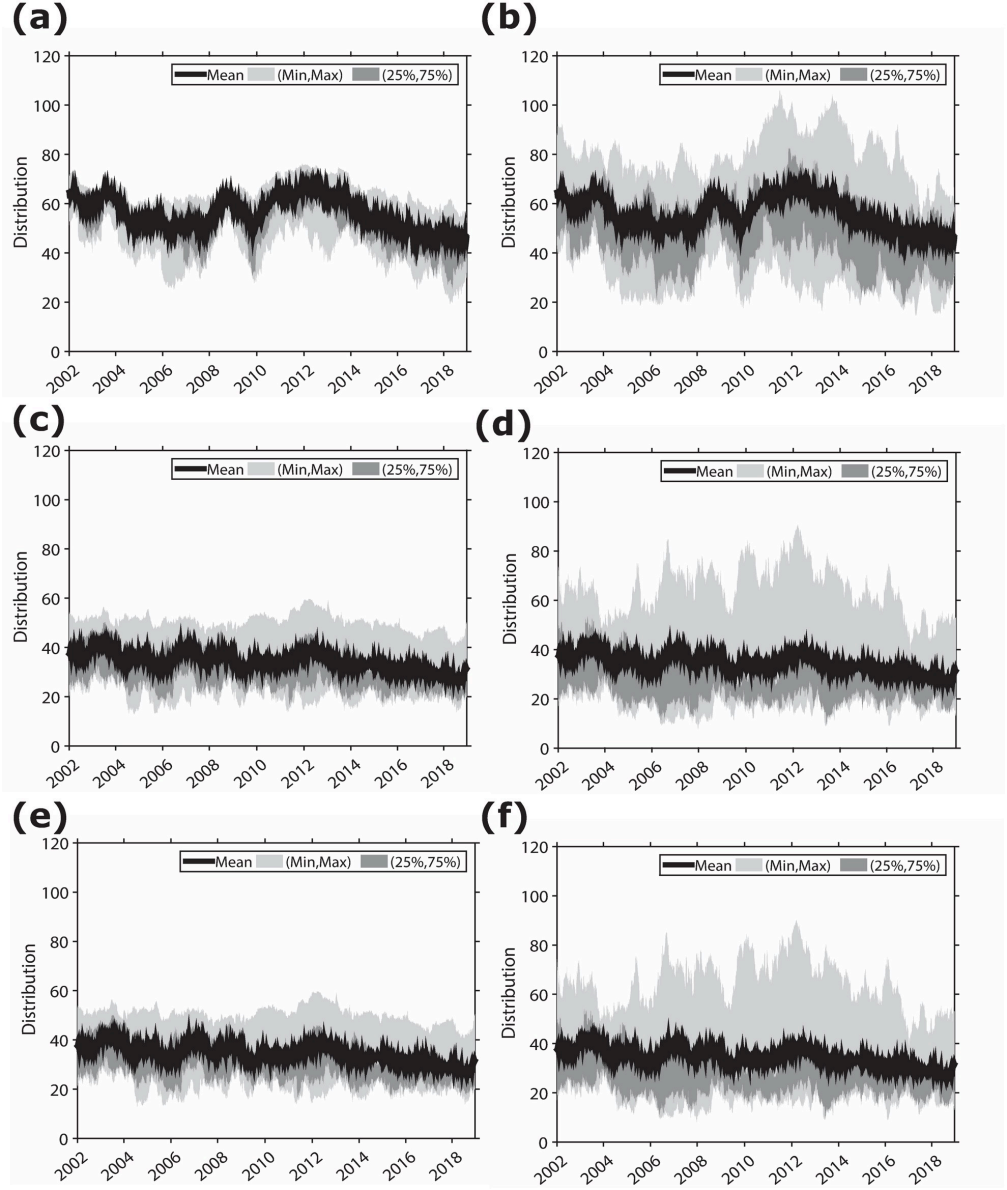
Rolling distribution of the total directional connectedness. ((a) and (b): “From” and “To” of TT, respectively; (c) and (d): “From” and “To” of NTI, respectively; and (e) and (f): “From” and “To” of NNTI, respectively). This figure plots the daily minimum, 25th percentile, mean, 75th percentile, and maximum values for the “To” and “From” distributions, representing the total directional connectedness of each investor group. The window width of the rolling estimation is 250 days.

Fig 4 illustrates the dynamics in the distribution of the connectedness with direction and weight, as measured for TT, NTI, and NNTI. The solid line represents the rolling distribution of the total directional connectedness each year. The figures on the left side, (a), (c), and (e), represent the inflows, and those on the right side, (b), (d), and (f), represent the outflows using TT, NTI, and NNTI, respectively. Different colors mean the range of 25% and 75% as well as the maximum and minimum values of connectedness. The purpose of displaying this distribution is to describe how much information investors share when economic events occur. During financial crises, the distribution of outflows of connectedness among investors is broader than that of inflows. This pattern implies that specific investors are more likely to disseminate negative shocks during an unstable market status, indicating that the investors play a pivotal role in the propagation of market-relevant information. Our findings suggest that our microscale approach to measuring connectedness effectively captures market conditions from the perspective of systemic risk [30]. In the following section, we explore identifying the specific investor groups that play a significant role in the formation and propagation of systemic risk.

### Identifying the source of destabilization

In this section, the study aims to identify the source of destabilization in the financial market by analyzing the dynamic connectedness among investors. This study used visualization, such as network diagrams, to represent the relationship between connectedness and stability in the financial market. [24] argue that information diffusion among the investor influences trading behavior and returns. We have a consistent view with the literature, and we would like to reveal the specific source of information propagation that contributes to market destabilization.

To identify the influence via investor activity in Total Connectedness, Table 4 presents the yearly average of the net value from the total connectedness matrix. Our findings highlight that retail investors play a significant role in the propagation of structural shocks in the stock market during the sample period. This result is in line with the findings of [40, 41], who suggest that retail investors may have psychological biases and be considered noise traders. Conversely, [42] provides that retail investors can contribute to providing liquidity and enhancing market efficiency through their individualized trading strategies. Our conjecture is that information transmitted from retail investors could affect financial market destabilization, especially during financial crises.

**Table 4:** The role of destabilization in total connectedness.

Panel A: Net value of each investor of Total Trading (TT)									
	FO	RE	BA	IS	IV	PF	FI	OF	OC
2002	-2.58	-12.36	4.96	-1.43	14.76	-0.22	12.13	-7.14	-8.11
2003	-7.55	-8.18	10.11	-0.15	13.95	6.60	-1.40	-6.46	-6.91
2004	-3.80	14.62	9.89	-8.57	2.72	1.22	5.41	-14.35	-7.16
2005	-8.73	9.51	11.55	-11.10	-0.93	9.65	-0.03	-18.02	8.11
2006	-5.79	6.55	0.95	-12.79	11.08	3.83	-1.83	-6.52	4.53
2007	4.19	7.40	1.40	-6.16	0.74	-4.82	17.44	-11.37	-8.81
2008	6.06	1.20	-1.87	-6.52	4.77	0.83	8.09	-10.56	-1.99
2009	-6.42	4.05	1.85	-8.18	3.70	-5.84	10.54	-7.15	7.45
2010	-4.60	4.22	-9.01	8.81	4.69	-3.85	16.94	-11.22	-5.97
2011	-11.49	12.21	1.92	6.70	4.57	-10.76	25.38	-17.54	-10.99
2012	4.40	11.62	-0.23	-1.38	13.34	-9.21	14.96	-19.82	-13.66
2013	-0.48	10.28	-1.96	-8.37	10.42	-5.31	25.33	-20.22	-9.70
2014	3.34	4.35	-5.34	-7.48	11.74	5.40	21.93	-21.68	-12.26
2015	-1.55	0.14	1.80	-1.04	21.70	-4.98	7.27	-8.15	-15.19
2016	-6.05	4.56	0.93	-2.85	16.95	-2.01	12.19	-13.72	-9.99
2017	0.84	0.35	0.78	-5.41	4.15	7.65	4.92	-4.64	-8.64
2018	-3.19	1.74	-1.80	-1.61	6.24	2.34	5.18	-5.48	-3.42
Panel B: Net value of each investor of Net Trading Imbalance (NTI)									
	FO	RE	BA	IS	IV	PF	FI	OF	OC
2002	-4.69	12.43	-7.06	-1.90	12.30	-1.28	2.53	-3.64	-8.69
2003	-1.40	5.13	-1.61	-5.44	9.35	-1.26	5.96	-10.67	-0.07
2004	-2.71	-0.36	-2.80	2.88	-1.02	10.50	0.53	-1.74	-5.29
2005	-6.48	10.28	-1.74	-1.29	2.23	10.36	-0.11	-2.24	-11.01
2006	-11.15	20.02	-7.87	-5.14	15.90	1.70	-8.11	-2.80	-2.55
2007	-5.77	21.35	-5.57	-7.36	15.07	4.76	-10.72	-7.60	-4.17
2008	-11.38	19.18	-3.29	-0.94	23.26	-0.85	-10.73	0.02	-15.28
2009	-5.73	14.94	-3.31	-9.29	15.34	-1.81	-7.99	-1.44	-0.71
2010	4.26	21.49	-2.64	-8.21	7.46	-9.56	-9.04	2.82	-6.58
2011	14.22	21.51	-0.55	-2.27	-8.81	-0.63	-5.89	-7.98	-9.60
2012	7.05	22.62	-1.76	-0.19	-11.26	1.72	-7.15	-5.67	-5.37
2013	7.52	19.89	-8.89	4.44	-14.27	1.20	-1.39	-8.77	0.27
2014	1.80	12.39	1.44	-2.96	-4.85	-4.01	4.38	0.92	-9.11
2015	3.58	11.53	-2.62	1.63	-9.37	-3.61	0.38	3.86	-5.39
2016	2.42	13.37	-2.27	-1.67	-3.11	4.14	-4.68	-6.61	-1.58
2017	0.61	6.03	-4.81	0.09	-0.98	1.99	0.92	-3.31	-0.55
2018	2.55	10.23	-0.31	-3.07	0.57	-1.32	-4.71	-5.93	1.98
Panel C: Net value of each investor of Normalized Net Trading Imbalance (NNTI)									
	FO	RE	BA	IS	IV	PF	FI	OF	OC
2002	-4.29	12.79	-6.89	-2.19	12.36	-1.34	2.33	-3.60	-9.18
2003	-1.29	5.33	-1.86	-5.40	9.27	-0.97	6.02	-11.05	-0.05
2004	-3.30	-0.23	-2.72	3.08	-0.89	10.54	0.86	-2.02	-5.33
2005	-6.58	10.07	-1.42	-0.85	1.97	10.07	-0.37	-2.17	-10.72
2006	-10.98	19.88	-7.48	-5.18	15.99	1.77	-8.19	-3.07	-2.76
2007	-5.87	21.80	-5.37	-7.45	15.04	4.55	-10.42	-7.86	-4.43
2008	-11.80	19.38	-3.48	-0.92	23.33	-0.85	-10.42	-0.06	15.17
2009	-5.77	14.78	-3.40	-9.32	15.13	-2.12	-7.42	-1.53	-0.35
2010	3.84	21.63	-2.79	-8.01	7.48	-9.22	-9.09	2.56	-6.40
2011	13.99	21.19	-0.48	-2.21	-8.77	-0.15	-5.93	-7.96	-9.68
2012	6.71	22.19	-1.65	-0.09	-11.02	2.19	-7.49	-5.77	-5.08
2013	7.88	20.20	-8.77	4.06	-14.09	1.26	-1.05	-9.66	0.16
2014	1.55	12.38	1.76	-2.99	-4.56	-4.09	4.69	0.60	-9.32
2015	3.31	11.54	-1.69	1.51	-9.31	-3.61	0.04	3.96	-5.75
2016	2.72	13.25	-2.52	-1.96	-3.38	4.09	-4.74	-6.06	-1.40
2017	0.46	5.58	-4.18	-0.06	-0.80	1.98	0.95	-3.89	-0.03
2018	2.51	10.11	-0.06	-3.14	0.32	-1.60	-4.58	-6.03	2.47

The table provides the yearly average of the net value from the connectedness matrix. Investors with a positive net value are defined as system sources and those with a negative net value are defined as system sinks. The nine types of investors are classified as follows: Foreign (FO), Retail (RE), Banks (BA), Insurance (IS), Investment (IV), Pension Funds (PF), Financial Investment (FI), Other Financial (OF), and Other Corporate (OC).

**Fig 5:**
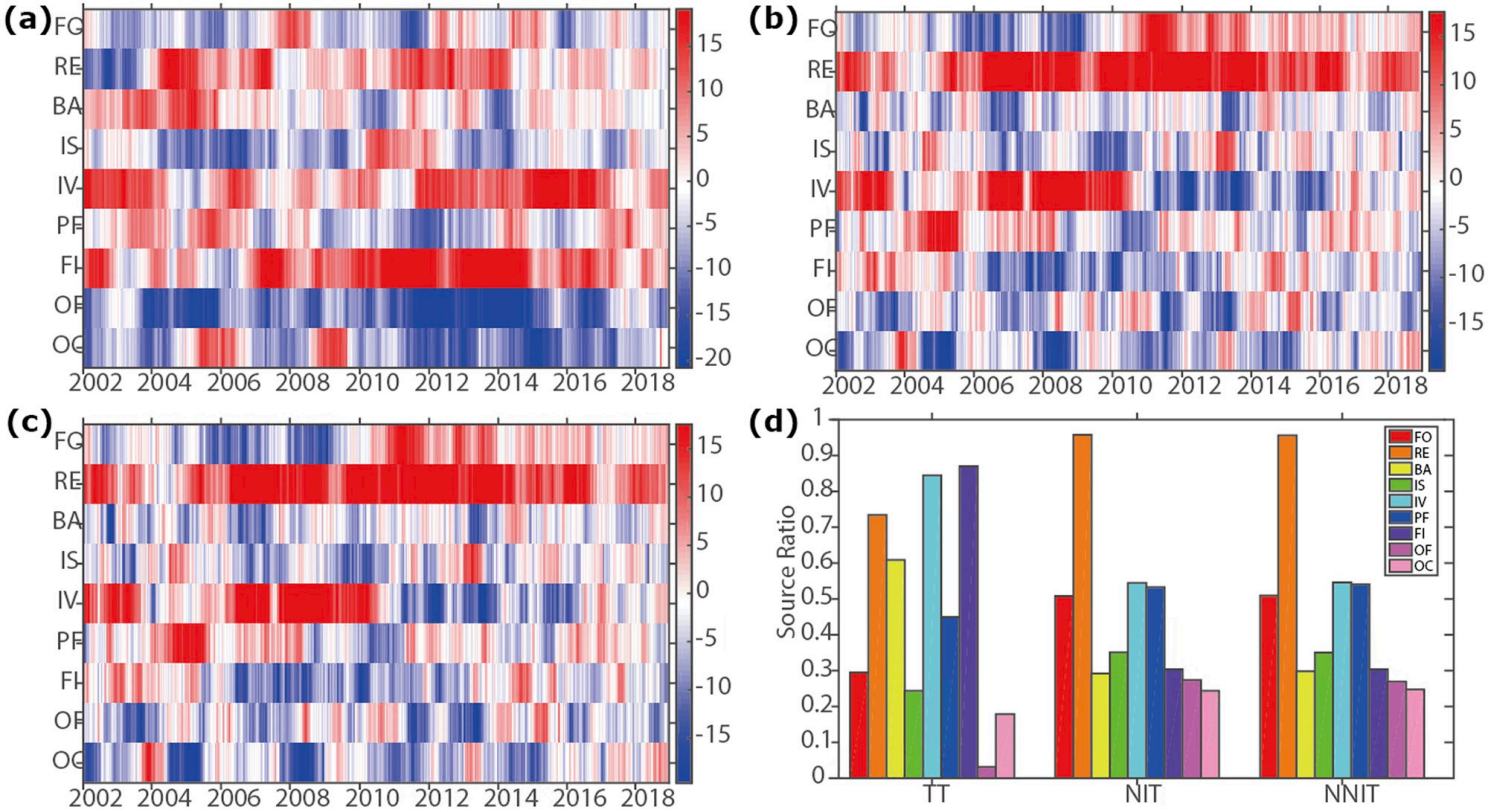
(Color online) Dynamics of Investor Sources and Sinks in Total Connectedness: (a) “Net” value for TT, (b) “Net” value for NTI, (c) “Net” value for NNTI, and (d) Source ratio for system sources across investor groups in each dataset. Investors with a positive net value are categorized as system sources, while those with a negative net value are termed as system sinks.

In addition, Fig 5 examines the roles of different investors as either sources or sinks in terms of connectedness, defined as those with positive and negative net values, respectively. Fig 5 suggests that the dynamics of sources and sinks account for destabilizing the financial market. Fig 5(a)–5(c) suggest that retail investors often act detrimentally to market stability, serving mainly as sources of information flow to other market participants each year. Specifically, Fig 5(a) shows that investment and financial institutions also play significant roles as system sources in the U.S. subprime crisis and the European debt crisis. Fig 5(b) and 5(c) indicate that the connectedness by sources, such as retail and investment, increases before the 2008 financial crisis. Last, Fig 5(d) quantifies the source ratio, defined as the ratio of time periods where they act as a source. These results indicate that retail investors have a significant role in the dissemination of information, thus impacting the stability of the financial market.

**Fig 6:**
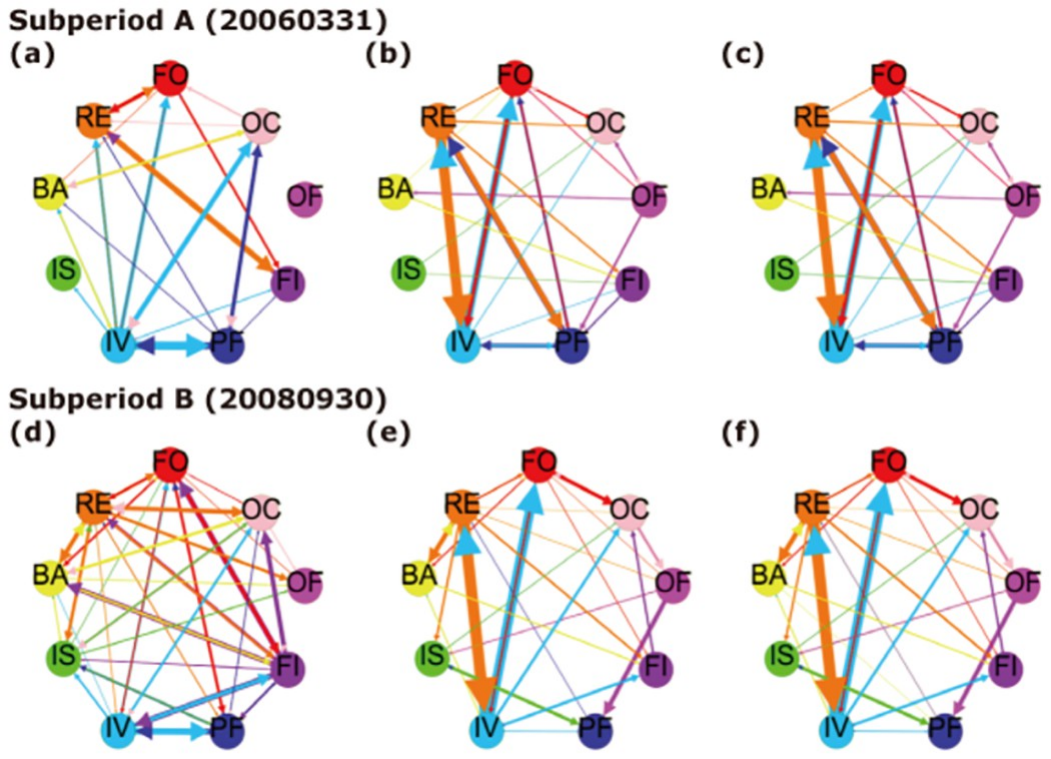
(Color online) investor network graph, periods of tranquil (20060331)-crisis (20080930). This figure plots the network of the pairwise connectedness among investor groups during both tranquil and crisis periods. (a), (b), (c) depict networks based on total trading, net trading imbalance, and normalized net trading imbalance, respectively, during a tranquil period. Similarly, (d), (e), and (f) show these networks during a crisis period. The nodes represent each investor group and the edges represent the directional connectedness between them. The nine types of investors are classified as follows: Foreign (FO), Retail (RE), Banks (BA), Insurance (IS), Investment (IV), Pension Funds (PF), Financial Investment (FI), Other Financial (OF), and Other Corporate (OC).

Fig 6 illustrates weighted and directed networks to display the structure of heterogeneous investors and their roles during a global financial crisis. In Fig 6, nodes are different investor groups, and links are pairwise directional connectedness between investor groups. The color of the node and width of the edge represent the strength of the connection between them. The networks are created by links that exceed a certain threshold of the connectedness, calculated as *μ* − *aσ*, where *μ* and *σ* denote the mean and standard deviation of each connectedness matrix at time *t*. The constant *a* is fixed at 0.3 for all datasets. This threshold aptly captures the magnitude of information flow between investor groups during tranquil and turbulent periods.

To capture the connectedness response at Lehman Brothers’ bankruptcy, announced on September 15, 2008, Fig 6 shows the network graphs on March 31, 2006, and on September 30, 2008. These graphs reveal that retail investors are robust transmitters of information to other investor groups in the KOSPI market. Fig 6(a)–6(c) have lower connectedness than Fig 6(d)–6(f), indicating that retail investors indiscriminately contributed to the spread of information across the stock market after the Lehman Brothers collapse.

These findings align with prior studies indicating that retail investors, often considered noise traders due to psychological biases [24], significantly contribute to the dynamics of the financial markets. Additionally, our results underscore an increase in connectedness among the primary sources of systemic risk preceding global financial crises, suggesting that this factor should be considered when evaluating contagion channels for exogenous shocks.

### Pricing the connectedness of shocks of total trading in the cross-section

In this subsection, we focus on evaluating the impact of change in total connectedness captured through total trading activities on expected returns. This is a critical step for quantifying the risk premium associated with market-wide connectedness and assess its pricing relevance. This is consistent with the view of [43], who provided a theoretical framework that utilizes asset pricing using a demand system approach. To achieve this, we employ a cross-sectional regression framework using 1-month excess returns as the dependent variable. We include the regression intercept from both the Capital Asset Pricing Model (CAPM) and the Fama-French three- and five-factor models as exploratory variables.

The quintile portfolios, classified according to their past-month *β*_Δ*TC*_ through Eq 8, are summarized in Table 5. We employed portfolio analyses based on Eqs 9 and 10, after controlling for the CAPM model or for Fama-French factors [44]. After controlling the Fama and French three and five factors, the result is consistent with that of the CAPM model. The columns marked “CAPM Alpha”, “FF-3 Alpha”, and “FF-5 Alpha” represent the time series alphas of the portfolios of the CAPM, FF-3 model, and FF-5 model, respectively. We reject the hypothesis that the ex post *β*_Δ*TC*_ loadings are equivalent to zero. Additionally, sorting stocks based on past *β*_Δ*TC*_ indicates that the returns of stocks are dependent on their sensitivities to differences in total connectedness among investor groups.

**Table 5:** Portfolio sorted by exposure to aggregate information shocks (total trading).

Rank	Mean	SD	Size	B/M	CAPM *α*	FF-3 *α*	FF-5 *α*	*β*
1	1.4472	6.7435	11.8960	6.9512	1.0006***	1.5017***	1.2279***	-3.3631
					[3.2303]	[3.0792]	[2.2316]	
2	1.6999	6.3612	11.9013	7.0937	1.2528***	1.5493***	1.3845***	-1.0228
					[4.6242]	[3.1310]	[2.6786]	
3	1.5438	6.0362	11.8601	7.1318	1.1247***	1.2997***	1.2191***	-0.0404
					[4.3034]	[2.7955]	[2.4265]	
4	1.4992	6.3185	11.9246	7.1068	1.0555***	1.2798***	1.2018***	0.9060
					[3.7818]	[2.6455]	[2.4167]	
5	1.3477	6.6658	11.8602	6.9570	0.8915***	1.2368***	1.1295**	3.0962
					[2.9726]	[2.4202]	[1.9136]	
5-1	-0.0995				-0.1091	-0.2649	-0.0984	
					[-0.5223]	[-1.2134]	[-0.4437]	

A value-weighted quantile portfolio is generated every month with a regression of excess individual stock returns on ΔTC to control the MKT factor as in Eq 8, using the previous month’s daily data. Stocks are sorted into quantiles 1 through quantiles 5 based on the coefficient of *β*_Δ*TC*_. Mean and Std. Dev. are measured as a percentage of the monthly excess returns. Size is defined by the natural logarithm of the market capitalization for firms within the portfolio, and B/M represents the book-to-market ratio. The row labeled 5-1 reports the difference between the monthly return of portfolio five and that of portfolio one. The t-statistics, which tested the null hypothesis that the average portfolio return is equal to zero, are shown in parentheses and adjusted using six lags following Newey and West (1987). The *α* column refers to Jensen’s *α* for the CAPM and the Fama-French (1993) 3-factor and 5-factor models. *β* represents a value-weighted beta included in each quantile portfolio at the beginning of the month.

** and *** indicate statistical significance at the 5% and 1% levels, respectively.

Our study finds significant variation in stock returns based on sensitivity to total connectedness, denoted as Δ*TC*. Stocks in the 1st quintile portfolio, which are less sensitive to systemic risk, exhibit higher mean returns than those in the 5th quintile portfolio, which are more sensitive to systemic risk. These findings are consistent with those of [37], who posited that stocks with higher exposure to market-wide risk factors tend to offer lower returns. This relationship supports that in the presence of market volatility, investors with risk aversion may adjust their investment opportunities based on their preferences for co-skewness. Specifically, stocks that are highly affected by changes in market volatility may be desirable to these investors, despite having low average returns [45]. This result also supports the findings of studies that have examined the risk-averse agents’ demand when increasing volatility under a heteroskedastic environment [46]. While our 5-1 row showed a statistically insignificant difference in returns, the overall pattern across the quintiles suggests a relationship that merits further study.

The importance of identifying and managing exposure to systemic risk cannot be overstated, as it can have significant implications for investment decisions and performance. Our study highlights the need for risk management practices that account for systemic risk and provides important insights for investors seeking to optimize their portfolio performance.

## Conclusion

This paper investigates systemic risk in the Korean stock market through an investor-based connectedness measure. We reveal that the systemic risk measure, based on investor activity, provides insights into market dynamics during both domestic and global financial crises. Furthermore, our findings affirm that retail investors play a substantial role in total connectedness and possess the potential to trigger contagion effects, in line with prior studies [32, 47, 48]. Our analysis also identifies alphas as a proxy of additional returns, indicating that the stocks with the low (high) sensitivity of connectedness beta are likely to outperform (underperform).

Despite these insights, our study has limitations that warrant further investigation. We do not directly capture investor sentiment, offering a more psychological perspective. Typically, it is measured using proxies such as news media or the trading volume of specific stocks. We only focus on a more structural view of market dynamics, emphasizing the influences among different investor groups. Additionally, the lack of statistically significant differences between the highest and lowest quintiles of connectedness beta in returns deserves further investigation. These limitations not only highlight the avenues for future research but also suggest the need for more comprehensive systemic risk measures, such as accounting for carbon risk, investor reactions to market events, or the shocks arising from technological developments. Understanding these elements could further refine portfolio optimization strategies and risk assessment.

The implications of our study are manifold for policy-makers and regulatory agencies. Our findings provide compelling evidence that systemic risk in the Korean stock market is significantly influenced by retail investor activity. By identifying which types of investors are significant contributors to risk propagation, regulators could make more informed decisions about where to direct their interventions. For instance, policies could be designed to mitigate the impact of retail trading on market stability during economic crises. Moreover, this paper suggests developing a complex and multidimensional model that could incorporate investor-based connectedness with other variables, such as connectedness among financial institutions and macroeconomic indices, to predict financial crises or assess systemic risk.
