# Correction: The contagion effect of heterogeneous investor groups

**DOI:** 10.1371/journal.pone.0352902

**Published:** 2026-06-29

**Authors:** A-Young Park, Gabjin Oh

The Acknowledgement section for this article is incorrect. The correct Acknowledgement section is: The authors thank seminar participants at the conference of 25th International Conference on Computing in Economics and Finance in Ottawa. This article is based in part on the first author’s doctoral dissertation, Essays on financial connectedness, systemic risk, and performance (Chosun University, 2021), and was later revised and developed for journal publication. The conclusions, views, opinions, and errors are those of the authors.
